# Molecular epidemiology and phylogeny of *Anaplasma* species in goats from Pakistan

**DOI:** 10.1371/journal.pone.0325467

**Published:** 2025-05-30

**Authors:** Sumaira Sumaira, Muneer Rehman, Arshad Hussain, Rashid Mehmood, Nimra Ashraf, Ioannis A. Giantsis, Waqas Umer, Ayesha Ayesha, Shakir Ullah, Adil Khan, Mourad Ben Said, Furhan Iqbal, Ayman A. Swelum

**Affiliations:** 1 Institute of Zoology, Bahauddin Zakariya University, Multan, Pakistan; 2 Department of Animal Science, Faculty of Agriculture, Forestry and Natural Environment, Aristotle University of Thessaloniki, Thessaloniki, Greece; 3 Department of Zoology, Abdul Wali Khan University Mardan Pakistan, Mardan, Pakistan; 4 Department of Zoology, Bacha Khan University, Charsadda, Khyber Pakhtunkhwa, Pakistan; 5 Laboratory of Microbiology, National School of Veterinary Medicine of Sidi Thabet, University of Manouba, Manouba, Tunisia; 6 Department of Basic Sciences, Higher Institute of Biotechnology of Sidi Thabet, University of Manouba, Manouba, Tunisia; 7 Department of Animal Production, College of Food and Agriculture Sciences, King Saud University, Riyadh, Saudi Arabia; Van Yuzuncu Yil University Faculty of Veterinary Medicine: Yuzuncu Yil Universitesi Veteriner Fakultesi, TÜRKIYE

## Abstract

Despite a goat population of approximately 80 million in Pakistan during 2020−2021, the prevalence of vector-borne pathogens in goats remains largely underexplored. This study aimed to assess the molecular prevalence and phylogenetic characteristics of *Anaplasma ovis, Anaplasma marginale* and *Anaplasma phagocytophilum* in goat blood samples (N = 239) collected from three districts (Muzaffargarh, Rajanpur, and Dera Ghazi Khan) in Punjab between September 2023 and October 2024. Blood samples were first screened with generic and then with species specific primers. Molecular analyses revealed a prevalence of 39% for *Anaplasma* spp. and 14% for *A. ovis*. *A. marginale* and *A. phagocytophilum* were not detected. DNA sequencing, by targeting 16S rRNA and *msp4* genes, and BLAST analysis confirmed the presence of *Anaplasma* spp. and *A. ovis,* respectively. For both screening, bacterial prevalence rates varied significantly across sampling sites (P = 0.01 for *Anaplasma* spp. and P = 0.04 for *A. ovis*). Additionally, the prevalence of *Anaplasma* spp. significantly differed among goat breeds (P = 0.004), while no association was found between goat sex and bacterial infections (P > 0.05 for both screening). Notably, *Anaplasma* spp. infection was associated with a significant decrease in red blood cell count and hemoglobin concentration, while *A. ovis* infection did not affect the complete blood count profile. Phylogenetic analysis revealed that our *Anaplasma* spp. isolates clustered with those from Iran, Cyprus and China while our *A. ovis* isolates clustered with those from Pakistan, China, and Sudan. In conclusion, this study reports the presence of *Anaplasma* spp. and *A. ovis* in Pakistani goats and recommends large-scale studies across diverse geo-climatic regions to further investigate the epidemiology, genetic diversity and host-parasite interactions for effective control of these infections in local goat populations.

## 1. Introduction

Pakistan is home to a significant population of sheep and goats, estimated at 109.4 million according to the 2019 animal census, making it the third-largest country in Asia for small ruminant populations [1]. Goats are particularly favored due to their resilience to harsh climatic conditions and drought, requiring minimal care [2]. Predominantly raised in rural areas, these animals serve as a vital source of income through the production of meat, milk, hair, and skins [3]. Over 35 goat breeds exist in Pakistan, with the Beetal, Nachi, Diara Din Panah, Damani, Kamori, and Kacchani recognized for their superior meat and milk yields [4].

However, diseases, particularly vector-borne infections, pose significant challenges for small ruminants, leading to considerable economic losses through animal morbidity and mortality [2]. The incidence of ticks is notably high in this region, as the sub-tropical climate provides optimal conditions for tick proliferation [5]. Anaplasmosis, a prominent tick-borne disease, affects a wide range of wild and domestic animals, especially small ruminants [6]. Goats are considered reservoir hosts for several *Anaplasma* species, including *A. marginale*, *A. phagocytophilum*, *A. ovis*, and *A. capra* [2]. Some of these species have zoonotic potential, posing health risks to humans. Various tick species, such as *Haemaphysalis longicornis*, *Ixodes persulcatus*, and *Rhipicephalus microplus*, are known to transmit *Anaplasma* to different hosts [7]. *A. ovis* is reported in the Mediterranean Basin, central Europe, and tropical and sub-tropical regions globally, primarily transmitted by ticks from the *Rhipicephalus* and *Dermacentor* genera. Major consequences of *A. ovis* infections include weight loss, decreased productivity, hemoglobinuria, hemolytic anemia, abortion, and in acute cases, death [8]. Treatment for anaplasmosis in small ruminants typically involves intramuscular administration of 20 mg/kg of oxytetracycline [9].

Despite the crucial role that goats play in the livelihoods of many people in rural Pakistan, they are infrequently screened for blood-borne bacterial pathogens. To fill this gap, blood samples were collected from goats across three districts in Punjab, Pakistan, and screened for the DNA of *Anaplasma* spp. by using generic primers as well as for the presence of *A. ovis. A. marginale* and *A. phagocytophilum* by using species specific primers during PCR followed by DNA sequencing for bacterial confirmation. Additionally, the study assessed risk factors associated with each infection, the phylogeny of these *Anaplasma* species, and the potential effects of each bacterium on the complete blood count of goats.

## 2. Materials and methods

### 2.1. Ethical approval and inclusivity in global research

Ethical Research Committee of the Bahauddin Zakariya University Multan (Pakistan) approved all the experimental procedures and protocols applied in this study via letter number BZU./ Ethics/23–22. Additional information regarding the ethical, cultural, and scientific considerations specific to inclusivity in global research is included in the S1 File.

### 2.2. Study area and blood sampling

**Fig 1:**
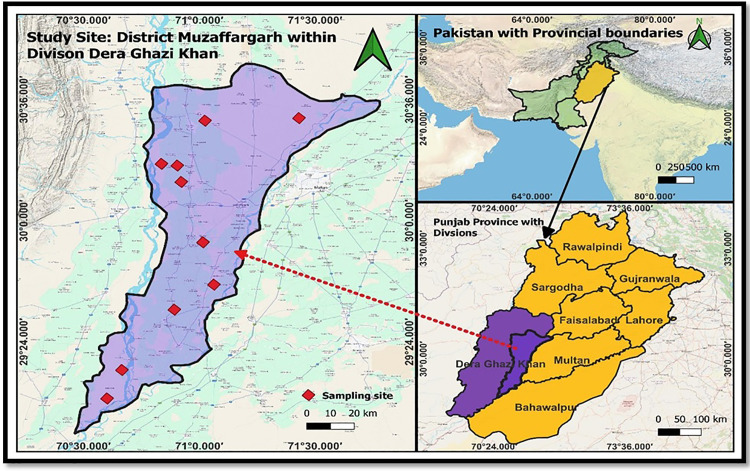
Map of Pakistan with marked Punjab province. In the magnified map of Punjab, the sampling districts that are highlighted from where the goat blood samples were collected during present Study.

An active epidemiological survey was conducted to determine the molecular prevalence of *Anaplasma* species in goat blood samples from three districts (Muzaffargarh, Rajanpur, and Dera Ghazi Khan) in Punjab, Pakistan. The geographical differences among the sampling areas led to the hypothesis of varying parasite prevalence (Fig 1). A total of 239 goats were enrolled from September 2023 to October 2024, with informed oral consent obtained from the owners. The sample included 40 goats from Muzaffargarh, 42 from Rajanpur, and 157 from Dera Ghazi Khan, representing 10 different breeds: Sindhi, Nachi, Makhi Cheena, Beetal, Nukri, Lailpuri, Teedy, Gulab Nukri, Desi, and Daira Din Panah. Approximately 2 mL of blood was collected from the jugular vein of each animal and preserved in a blood collection tube containing 0.5 M EDTA as an anticoagulant. A questionnaire was completed at the sampling site with the assistance of goat owners to gather epidemiological data on the prevalence of *A. capra* and *A. ovis* among the enrolled goats.

### 2.3. Complete blood count analysis

Hematological parameters including white blood cell count, monocytes (%), red blood cells, hemoglobin, hematocrit (%), mean cell volume, mean corpuscular hemoglobin, and eosinophils (%) were analyzed using an automated hematological analyzer (Mythic TM 18 Vet, Orpheus, Switzerland) in all goat blood samples collected during the present study.

### 2.4. DNA extraction and detection of pathogens by PCR

DNA extraction from blood samples was performed using an inorganic method, as previously described by Aziz et al. [2]. The extracted DNA was confirmed via agarose gel electrophoresis and then screened for the presence of *Anaplasma* spp., *A. ovis. A. marginale* and *A. phagocytophilum* by targeting the 16S rRNA, *msp4, msp1b* and *msp2* partial sequences, respectively, using previously reported primers and protocols [10–13] (S1 Table in S1 File). Amplification was carried out with a GeneAmp® PCR System 2700 (Applied Biosystems Inc., UK). For each PCR reaction, positive controls included the available *A. ovis. A. marginale* and *A. phagocytophilum* DNA in our lab from previous projects> Double-distilled water instead of DNA was used as a negative control.

### 2.5. DNA sequencing and phylogenetic analysis

Positive PCR products were sent to a commercial service provider (First Base Malaysia) for confirmation through Sanger sequencing. The sequences were obtained in ab1 format from the sequence provider that was used in FinchTV (version 1.4.0) to remove any low-quality nucleotide at both ends of the sequence. The remaining clean sequences were saved in FASTA format and used in BLAST analysis and subsequently submitted to GenBank. For both bacteria, similar sequences were downloaded from BLAST output to be used in phylogenetic analysis. These sequences were first aligned by using ClustalW multiple sequence alignment followed by substitution model selection using BIC and AIC values of the MEGA’s integrated model selection tool. Finally, the phylogenetic tree was inferred using Maximum Likelihood method with 1000 bootstrap iteration. *Rickettsia hoogstraalii* was utilized as outgroup in the phylogenetic analysis.

### 2.6. Statistical analysis

Statistical analysis was conducted with the Statistical package Minitab (Minitab, USA). Significance level was set at P ≤ 0.05. Fischer exact test was calculated to correlate the bacterial prevalence with the studied epidemiological factors. One way ANOVA was used to compare parasite prevalence between sampling sites and goat breeds. A two-sample t-test was conducted to compare the complete blood count parameters between the positive and negative groups for *Anaplasma* species.

## 3. Results

### 3.1. *Molecular epidemiology of Anaplasma* spp

PCR amplification of a 345 base pair fragment from the 16S rRNA gene of *Anaplasma* spp. was successful in 94 out of 239 goat blood samples (39.3%) collected from three districts in Punjab. *Anaplasma* spp. was detected in goats from all districts included in the study. One-way ANOVA revealed significant variation in prevalence among the sampling districts (P = 0.01), with the highest infection rates observed in Muzaffargarh (55%), followed by Rajanpur (50%) and Dera Ghazi Khan (32%) (Table 1). All enrolled goats from 10 different breeds tested positive for *Anaplasma* spp. One-way ANOVA results indicated significant differences in bacterial prevalence among goat breeds (P = 0.004), with the highest prevalence in Sindhi (100%), followed by Nachi (67%), Teddy (61%), Lailpuri (60%), Desi (58%), Makhi Cheena (43%), Beetal (38%), Gulab Nukri (35%), Nukri (28%), and Dera Din Panah (27%) (Table 2). Notably, there was no association between bacterial prevalence and the sex of the enrolled goats (P = 0.1) (S2 Table in S1 File).

**Table 1:** Overall comparison of *Anaplasma* spp. and *A. ovis* prevalence among the goats enrolled from three districts in Punjab during the present study. % prevalence is given in parentheses. P-value represents the output of one -way ANOVA test.

Sampling district	*Anaplasma* spp. + ve	*Anaplasma* spp. -ve	P value	*Anaplasma ovis* +ve	*Anaplasma ovis* -ve	P value
Dera Ghazi Khan	51/157 (32%)	106/157 (68%)		18/157 (12%)	139/157 (88%)	
Rajanpur	21/42 (50%)	21/42 (50%)	0.01**	11/42 (26%)	31/42 (74%)	0.04*
Muzaffargarh	22/40 (55%)	18/40 (45%)		04/40 (10%)	36/40 (90%)	
**Total**	**94/239 (39%)**	**145/239 (61%)**		**33/239 (14%)**	**206/239 (86%)**	

P < 0.05 = Significant (*); P ≤ 0.01 = Significant (**).

**Table 2:** Prevalence of *Anaplasma* spp. and *A. ovis* among the screened goat breeds enrolled from three sampling districts in Punjab during the present study. % prevalence of each pathogen is given in parentheses. P-value represents the results of the one way ANOVA test.

Breed	*Anaplasma* spp. + ve	*Anaplasma* spp. - ve	P value	*Anaplasma ovis* +ve	*Anaplasma ovis* -ve	P value
Daira Din Panah	21/78 (27%)	57/78 (73%)		09/78 (12%)	69/78 (88%)	
Nukri	11/40 (28%)	29/40 (72%)		03/37 (08%)	34/37 (92%)	
Desi	19/33 (58%)	14/33 (42%)		05/33 (15%)	28/33 (85%)	
Teddy	14/23 (61%)	09/23 (39%)		05/23 (22%)	18/23 (78%)	
Makhi Cheena	09/21 (43%)	12/21 (57%)		01/21 (05%)	20/21 (95%)	
Gulab Mukri	06/17 (35%)	11/17 (65%)	0.004**	04/17 (24%)	13/17 (76%)	**0.6**
Beetal	05/13 (38%)	08/13 (62%)		02/13 (15%)	11/13 (85%)	
Lailpuri	06/10 (60%)	04/10 (40%)		03/10 (30%)	07/10 (70%)	
Nachi	02/03 (67%)	01/03 (33%)		0/03 (0%)	03/03 (100%)	
Sindhi	01/01 (100%)	0/01 (0%)		01/01 (100%)	0/01 (0%)	
**Total**	**94/239 (39%)**	**145/239 (61%)**		**33/239 (14%)**	**206/239 (86%)**	

P > 0.05 = Non significant; P < 0.01 = Significant (**).

### 3.2. *Potential impact of Anaplasma* spp. *infection on complete blood count parameters*

Analysis of complete blood count parameters revealed significant decreases in monocytes (%), red blood cell count, and hemoglobin concentration in goats infected with *Anaplasma* spp. compared to non-infected goats (P = 0.01 for both parameters) (Table 3). Specifically, the percentage of monocytes was lower in infected goats (1.76 ± 0.12%) than in those without infection (1.70 ± 0.07%), while the red blood cell count was significantly reduced from 2.94 ± 0.5 × 10^6^/µL in non-infected goats to 2.13 ± 0.14 × 10^6^/µL in infected ones. Hemoglobin concentration also showed a significant decline, with infected goats having a mean of 8.01 ± 0.32 g/dL compared to 8.81 ± 0.4 g/dL in non-infected goats (Table 3).

### 3.3. *Phylogenetic analysis of Anaplasma* spp. *based on 16S rRNA partial sequence*

**Fig 2:**
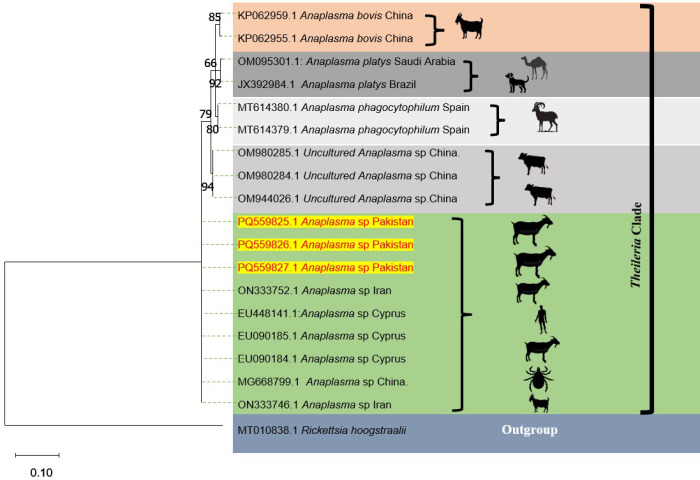
Phylogenetic analysis of *Anaplasma* spp. based on the partial 16S rRNA gene sequence. Sequences in highlighted in red were generated during present investigation.

Three partial 16S rRNA gene fragments from *Anaplasma* spp. positive PCR were confirmed by DNA sequencing and deposited in GenBank under accession numbers PQ559825, PQ559826, and PQ559827. BLAST analysis showed that the amplified sequences were 97–98% similar to previously deposited *Anaplasma* spp. sequences in GenBank. Phylogenetic analysis indicated that the Pakistani isolates clustered together and shared similarities with 16S rRNA sequences of *Anaplasma* spp. from ticks, human, small and large ruminants in Iran (ON333746 and ON333752), China (MG668799, OM980284, OM980285 and OM944026) and Cyprus (EU090184, EU090185 and EU448141). Using generic primers targeting the conserved 16S rRNA gene, the isolates also exhibited resemblances to *A. phagocytophilum* sequences amplified in wild ruminants in Spain, as well as to *A. platys* isolated from camel and dogs in Saudi Arabia, and Brazil respectively. Additionally, the isolates showed similarities with *A. bovis* detected in small ruminants from China (Fig 2).

### 3.4. Molecular epidemiology of A. ovis

PCR amplification yielded a 347 base pair fragment specific for the *msp4* gene of *A. ovis* in 33 out of 239 goat blood samples (14%) collected from three districts in Punjab province, Pakistan. Analysis indicated that *A. ovis* was present in infected goats from all three districts. One-way ANOVA revealed significant variation in *A. ovis* prevalence among the sampling districts (P = 0.04), with the highest infection rate found in goats from Rajanpur (26%), followed by Dera Ghazi Khan (12%) and Muzaffargarh (10%) (Table 1). During this study, goats from 10 different breeds were enrolled, and nine tested positive for *A. ovis*. However, one-way ANOVA indicated that bacterial prevalence did not vary significantly among the screened goat breeds (P = 0.600) (Table 2). Additionally, no association was found between bacterial prevalence and the sex of the enrolled goats (P = 0.1) (S2 Table in S1 File).

### 3.5. Potential impact of A. ovis infection on complete blood count parameters

Analysis of complete blood count parameters indicated that all measured values did not vary significantly (P > 0.05) between *A. ovis*-infected and uninfected goats enrolled from the Dera Ghazi Khan district during this study (Table 3). This includes white blood cell count, monocyte percentage, red blood cell count, and hemoglobin concentration, reflecting no notable impact of *A. ovis* infection on these hematological parameters (Table 3).

**Table 3:** Comparison of the studied complete blood count parameters between *Anaplasma* spp. and *A. ovis* positive and negative goats enrolled from Dera Ghazi Khan district in Punjab. Data is represented as mean ± standard error of mean. P-value indicates the result of two-sample t test calculated for each studied parameter.

Breed	*Anaplasma* spp. + ve	*Anaplasma* spp. –ve	P value	*Anaplasma ovis* +ve	*Anaplasma ovis* –ve	P value
White blood cell (x 10^3^/uL)	15.34 ± 0.7	14.98 ± 0.7	0.7	14.72 ± 1.3	15.15 ± 0.56	0.7
Monocytes (x 10^9^/L)	17.83 ± 3.9	13.9 ± 2.4	0.4	17.44 ± 3.5	16.9 ± 1.7	0.6
Monocytes (%)	1.76 ± 0.12	1.7 ± 0.07	0.01**	1.72 ± 0.24	1.66 ± 0.08	0.8
Red blood cells (x 10^6^/uL)	2.13 ± 0.14	2.94 ± 0.5	0.01**	14.72 ± 1.3	15.15 ± 0.56	0.4
Hemoglobin (gd/L)	8.01 ± 0.32	8.81 ± 0.4	0.01**	8.49 ± 0.70	8.56 ± 0.29	0.9
Hematocrit (%)	21.60 ± 01.2	01.2 ± 02.5	0.1	23.1 ± 2.5	23.8 ± 1.2	0.8
Mean cell volume (fL)	101.0 ± 2.2	102.7 ± 1.3	0.5	101.6 ± 2.9	102.2 ± 1.2	0.8
EOS %	01.53 ± 0.09	1.46 ± 0.06	0.5	01.44 ± 0.17	1.47 ± 0.06	0.9
Mean corpuscular hemoglobin (pg)	4.08 ± 1.9	40.9 ± 1.0	0.9	41.92 ± 1.8	40.7 ± 0.99	0.6

P > 0.05 = Non significant; P < 0.01 = Significant (**).

### 3.6. Phylogenetic analysis of Anaplasma ovis based on msp4 partial sequence

**Fig 3:**
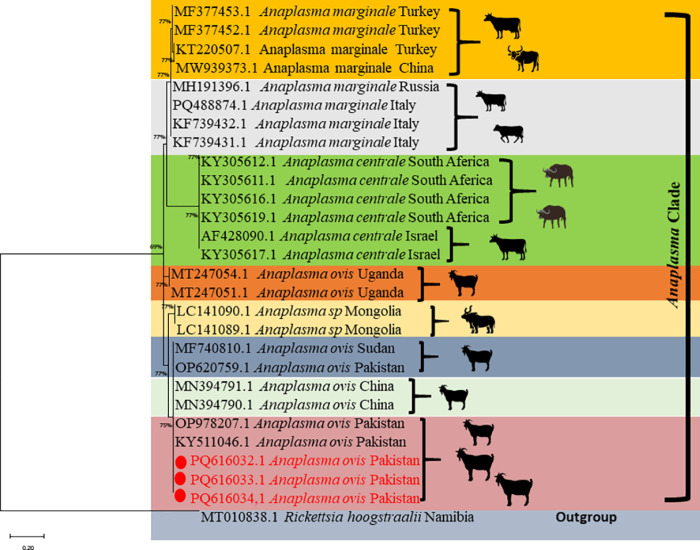
Phylogenetic tree of *Anaplasma ovis* based on the partial *msp4* gene sequences available in GenBank. The three haplotypes generated in this study are highlighted in red.

Three randomly selected *A. ovis*-positive PCR products (one from each district) were confirmed by DNA sequencing and deposited in GenBank with accession numbers PQ616032, PQ616033, and PQ616034. The Pakistani isolates clustered together, showing genetic similarity with *msp4* gene sequences of *A. ovis* previously reported from Pakistan (OP978207, KY511046, and OP620759), as well as from China (MN394790 and MN394791), Sudan (MF740810), Mongolia (LC141089), and Uganda (MT247051 and MT247054). The *msp4* gene sequences from Pakistani *A. ovis* were distinct from those of *A. marginale* reported from Turkey, China, Russia, and Italy, and differed from *A. centrale* sequences deposited from South Africa and Israel (Fig 3).

### 3.7. *Molecular epidemiology of A. marginale* and *A. phagocytophilum*

None of the screened goat samples were found infected with either *A. marginale* or *A. phagocytophilum* during present investigation.

## 4. Discussion

In Pakistan, goats are often referred to as the “poor man’s cow” in rural and less developed regions due to their adaptability to various living conditions and low raising costs [14]. Their frequent exposure to the environment increases the risk of infections, making them reservoirs for various infectious agents, including bacteria and protozoa [9]. *Ixodes* ticks are known vectors for transmitting *Anaplasma* species to a wide range of domestic and wild animals. The movement of small ruminants between summer and winter pastures is common in Pakistan, and these transhumant herds contribute to the spread of ticks and tick-borne pathogens. However, research addressing these issues remains limited in many countries, including Pakistan [15]. This investigation aimed to screen goat blood samples collected from three districts in Punjab for the presence and genetic diversity of various *Anaplasma* species.

In this study, we screened all the goat blood samples for *Anaplasma* species and found a relatively higher bacterial infection (39.3%) among them. As the screening was done by using generic primers, so more than one bacterial species were expected during this investigation. Hence, we used the amplicons from the 16S rRNA gene of *Anaplasma* spp. in the phylogenetic analysis. The nucleotide sequences amplified showed similarities to the 16S rRNA gene sequence of *Anaplasma* spp. reported in ruminants from Iran (unpublished data), Ixodidae ticks, goats and large ruminants (unpublished data) from China and goat and sheep [16] and infants in in Cyprus [17]. Future studies detecting these pathogens in goats would benefit from comparing the genetic diversity of *Anaplasma* spp. among goats from different regions of Pakistan.

In our study, we found that *Anaplasma* spp. prevalence varied significantly between sampling sites and goat breeds, but bacterial infection was not influenced by goat sex. In contrast, Razzaq et al. [9] reported that *Anaplasma* spp. infection was more prevalent in younger animals and nannies than in older goats and bucks. They also found that tick-infested animals had higher bacterial positivity rates than tick-free goats. Supporting our findings, Lin et al. [18] noted a significantly higher *Anaplasma* species infection rate in goats and ticks screened from Guizhou compared to those from Shandong province in China. Seo et al. [19] documented that *Anaplasma* species infection in goats from Korea did not vary with the sampling season; however, the infection rate for anaplasmosis was significantly higher in mixed grazing-confined farms compared to conventional and confined farms. Meanwhile, Peng et al. [20] found higher infection rates in goats older than one year and in grazing animals compared to those fed in households in China. This diverse data on risk factors underscores the need for more epidemiological studies to better understand the interactions of *Anaplasma* spp. with host goats.

A complete blood count is a crucial extension of the physical examination in small ruminants, aiding in the diagnosis of various diseases when clinical findings are unclear. This analysis is also valuable for establishing prognosis in many cases [2]. During present investigation, we observed a significant reduction in red blood cell count and hemoglobin concentration in *Anaplasma* spp. infected goats, indicating a hemolytic anemic condition. This observation aligns with previous findings that *Anaplasma* spp.-infected erythrocytes are destroyed by macrophages, leading to mild to severe hemolytic anemia [21].

During this study, we found that 39% of the screened goats had *Anaplasma* spp. infection and when we screened these infected goats for specific bacterial species, we fount that prevalence of *A. ovis* among these goats was 14%. We were unable to find the DNA of *A. marginale* and *A. phagocytophilum* among the screened goats. Our results indicate that probably some other *Anaplasma* species is circulating among the local goat populations that need to be identified in future studies. Several reports from Pakistan have explored the presence of this bacterium. Niaz et al. [22] reported 21% prevalence in small ruminants from four districts (Malakand, Swat, Bajaur, and Shangla) in Northern Pakistan. Ghaffar et al. [6] found a prevalence of 25.3% in goats from five tribal districts: Bajaur, Khyber, Mohmand, North Waziristan, and Orakzai in Khyber Pakhtunkhwa. Taqddus et al. [5] reported that 15% of goats from four districts (Layyah, Lohdran, Dera Ghazi Khan, and Rajanpur) in Punjab were positive for *A. ovis*. Additionally, Naeem et al. [1] documented a 12.5% infection rate in sheep from the Dera Ghazi Khan District in Punjab.

Numerous reports describe *A. ovis* infection in goats from various countries. Prevalence rates include 78.8% in goats from Kurdistan province [8], 76% in Botswana [23], 70.3% in Tunisia [24], 69% in Mongolia [25], 54.5% in China [26], 54% in the suburbs of Ahvaz in Iran [27], 52% in France [28], 34.2% in Kenya [29], 29.7% in Turkey [30], 14.8% in Bangladesh [31], and 1.5% in Thailand [32]. The variability in the prevalence of this bacterium across different studies may be attributed to differing geo-climatic conditions, male to female ratios, immune status of host animals, tick density, and farm management practices in the respective study areas [1].

Identifying tick-borne bacteria based on their taxonomic classification is crucial for developing effective therapeutic approaches for their control [33]. However, there are only a few reports from Pakistan regarding the genetic diversity of *A. ovis* detected in small ruminants. Therefore, the three amplified PCR products from the *msp4* gene in this investigation were utilized for the phylogenetic analysis of this bacterium. The *msp4* sequences amplified in this study were similar to those of *A. ovis* isolated from small ruminants in Pakistan [1,34], as well as from yak and sheep in China [35], cattle in Sudan [36], livestock from Mongolia [37], and ruminants from Uganda (unpublished data). The three *msp4* isolates generated in this study clustered together with previously reported *msp4* sequences of *A. ovis* detected in Pakistani ruminants, indicating that a single bacterial strain is prevalent among local ruminants (Fig 3). Further studies examining the *msp4* genetic diversity of *A. ovis* in animals from various regions of Pakistan are needed to elucidate the genetic diversity of this bacterium.

In the present study, we found that *A. ovis* infection varied significantly between sampling sites, but goat breed and sex showed no association with bacterial infection. Our results align with those of Taqddus et al. [5], who also observed significant variation in *A. ovis* prevalence in goats from four districts in Punjab, with the highest infection rate detected in Layyah and the lowest in Lohdran. This suggests that geo-climatic conditions and animal management practices vary across districts in Punjab, impacting bacterial prevalence. In contrast, Niaz et al. [22] identified goat age, grazing system, and acaricide treatment as significant determinants of *A. ovis* infection in Khyber Pakhtunkhwa. Additionally, Abbas Zadeh et al. [27] recently reported that the Najdi goat breed in Iran exhibited a significantly higher *A. ovis* infection rate compared to the black breed, and they observed higher infection rates in goats over one year old than in those under one year. Ghaffar et al. [6] found that tick infestation in Pakistani goats was a significant predictor of *A. ovis*. Understanding the epidemiology of these infections is crucial for developing effective control measures and guiding future research aimed at improving goat health and productivity.

*A. ovis* is an intracellular bacterium that infects red blood cells of the host and typically causes anemia [24]. However, our results did not align with this general observation, as no significant changes in the complete blood count parameters were observed when comparing *A. ovis*-infected and uninfected goats in this study. One potential reason for this could be the inclusion of apparently healthy and asymptomatic animals, which may have resulted in insufficient bacterial load in the goat blood samples to induce significant changes in the hematological profile. Our findings are consistent with those of Abbas Zadeh et al. [27] and Cabezas-Cruz et al. [28], who also reported no significant differences in hematological parameters between healthy and *A. ovis*-infected goats from Iran and France, respectively. In contrast, Rahravani et al. [8] found a significant decrease in white blood cell count in *A. ovis*-infected goats from western Iran. They noted disturbances in mean cell volume, mean cell hemoglobin, mean cell hemoglobin concentration, and platelet counts in goats positive for the bacterial infection. Similarly, Ghaffar et al. [6] documented decreases in hematological parameters, including red blood cells, packed cell volume, hemoglobin, white blood cells, monocytes, granulocytes, lymphocytes, and platelet counts in *A. ovis*-positive animals.

## 5. Conclusion

This study reports a high prevalence of *Anaplasma* spp. and *A. ovis* infections in goats from Punjab, Pakistan. The infection rate of *Anaplasma* spp. varied between sampling districts and goat breeds, significantly affecting the complete blood count profile of the hosts. In contrast, *A. ovis* infection varied significantly between sampling sites but not among different goat breeds. Overall, the findings contribute to the growing body of knowledge on tick-borne diseases affecting small ruminants in Pakistan. The high prevalence of these pathogens underscores the need for large-scale surveillance studies in Pakistan to assess their status at the human-animal interface. Furthermore, it is essential to develop preventive and control strategies to mitigate the economic losses associated with anaplasmosis in small ruminants.

## Supporting information

S1 File All the methods were performed in accordance with ARRIVE guidelines laws and regulations. (DOC)
